# Precision estimates of relative and absolute cerebral blood flow in Alzheimer’s disease and cognitively normal individuals

**DOI:** 10.1177/0271678X221135270

**Published:** 2022-10-21

**Authors:** Fiona Heeman, Denise Visser, Maqsood Yaqub, Sander Verfaillie, Tessa Timmers, Yolande AL Pijnenburg, Wiesje M van der Flier, Bart NM van Berckel, Ronald Boellaard, Adriaan A Lammertsma, Sandeep SV Golla

**Affiliations:** 1 Department of Radiology and Nuclear Medicine, Amsterdam Neuroscience, Amsterdam UMC, Vrije Universiteit Amsterdam, Amsterdam, Netherlands; 2 Department of Medical Psychology, Amsterdam Public Health, Amsterdam UMC, Universiteit van Amsterdam, Amsterdam, Netherlands; 3 Alzheimer Center Amsterdam, Department of Neurology, Amsterdam Neuroscience, Amsterdam UMC, Vrije Universiteit Amsterdam, Amsterdam, The Netherlands; 4 Department of Epidemiology and Biostatistics, Amsterdam UMC, Vrije Universiteit Amsterdam, Amsterdam, The Netherlands

**Keywords:** Cerebral blood flow, PET, precision, Alzheimer’s disease, healthy controls

## Abstract

Alzheimer’s disease is characterized by regional reductions in cerebral blood flow (CBF). Although the gold standard for measuring CBF is [^15^O]H_2_O PET, proxies of relative CBF, derived from the early distribution phase of amyloid and tau tracers, have gained attention. The present study assessed precision of [^15^O]H_2_O derived relative and absolute CBF, and compared precision of these measures with that of (relative) CBF proxies. Dynamic [^15^O]H_2_O, [^18^F]florbetapir and [^18^F]flortaucipir PET test-retest (TrT) datasets with eleven, nine and fourteen subjects, respectively, were included. Analyses were performed using an arterial input model and/or a simplified reference tissue model, depending on the data available. Relative CBF values (i.e. *K*_1_/*K*_1_′ and/or *R*_1_) were obtained using cerebellar cortex as reference tissue and TrT repeatability (i.e. precision) was calculated and compared between tracers, parameters and clinical groups. Relative CBF had significantly better TrT repeatability than absolute CBF derived from [^15^O]H_2_O (*r* = −0.53), while best TrT repeatability was observed for [^18^F]florbetapir and [^18^F]flortaucipir *R*_1_ (*r* = −0.23, *r* = −0.33). Furthermore, only *R*_1_ showed, better TrT repeatability for cognitively normal individuals. High precision of CBF proxies could be due to a compensatory effect of the extraction fraction, although changes in extraction fraction could also bias these proxies, but not the gold standard.

## Introduction

In Alzheimer’s disease (AD), regional reductions in cerebral blood flow (CBF) have been reported in the temporal, parietal and posterior cingulate regions,^1–3^ predominantly in the later phases of the disease.^4,5^ As perfusion is tightly coupled to metabolism,^6^ CBF measures may have clinical relevance in terms of determining disease severity as an indirect marker of neurodegeneration or for differential diagnosis.^5,7,8^ Moreover, these reductions in perfusion have been linked to cognitive decline in AD.^9^

The gold standard technique for measuring CBF is [^15^O]H_2_O positron emission tomography (PET). However, the short half-life of oxygen-15 (122 sec) restricts its application to a few specialised PET centres equipped with an on-site cyclotron. During the last decade, several studies have evaluated whether a proxy of CBF can be derived from the early distribution phase of PET tracers that measure amyloid-β (Aβ) or tau burden.^7,9–12^ Promising results from studies investigating these proxies of relative CBF (i.e. *R*_1_ or the early frame standardized uptake value ratio) have led to an increased interest in so called dual-phase or dual-time window acquisition protocols for these tracers, given that they allow for extracting two different biomarkers.^12–15^ Apart from these PET-based methods, many other techniques exist for measuring CBF, such as arterial spin labelling (ASL) MRI, which has commonly been used in AD research.^16^

During the last decade, longitudinal PET studies have become increasingly prevalent in AD research, which emphasizes the importance of understanding the intrinsic variability of CBF measures to determine what magnitude of change signifies an actual change. Repeatability of [^15^O]H_2_O PET derived absolute CBF measures has previously been characterized^17,18^ and more recently, test-retest (TrT) repeatability has been characterized for a global cortical volume of interest (VOI) for [^18^F]florbetapir derived *R*_1_ and across various ROIs for [^11^C]PiB derived *R*_1_.^19,20^ The repeatability for these CBF proxies was substantially better than TrT repeatability of [^15^O]H_2_O PET derived absolute CBF measures.^17^ It has been hypothesized that the lower TrT repeatability of the absolute CBF measures is mainly due to large global variations in CBF, which are normalized when calculating a relative measure of CBF. To contribute to the understanding of repeatability of CBF measures, the main purpose of the present study was to compare repeatability of [^15^O]H_2_O PET derived relative and absolute CBF measures. In addition, repeatability of these [^15^O]H_2_O PET derived measures was also compared with that of relative CBF proxies (i.e. relative tracer delivery *K*_1_/*K*_1_′ and/or *R*_1_) estimated from [^18^F]florbetapir and [^18^F]flortaucipir scans across a range of VOIs. A secondary aim was to assess whether repeatability of relative CBF proxies was stable across diagnostic groups for [^18^F]florbetapir and [^18^F]flortaucipir.^9,21^

## Materials and methods

### Participants & image acquisition

The following three separate datasets were retrospectively included. Before participating in the study, all participants provided written informed consent in accordance with the Declaration of Helsinki. Study protocols were approved by the local Medical Ethics Review Committee of the Amsterdam UMC, VUmc and in case of the [^15^O]H_2_O study, also by the local Medical Ethics Review Committee of the Amsterdam UMC, AMC.

#### [^15^O]H_2_O dataset

Eleven healthy control participants underwent repeated dynamic [^15^O]H_2_O PET scanning with arterial blood sampling (described below) on the same day.^22^ Briefly, a low-dose CT was acquired for attenuation and scatter correction purposes and then, a 10 min dynamic PET scan was performed starting at tracer injection. PET scans were acquired on a Philips Gemini TF-64 PET/CT system (Philips Healthcare, Cleveland, TN, USA) and reconstructed into 25 frames of progressively increasing duration using the row action maximum likelihood algorithm (LOR-RAMLA) reconstruction algorithm, with the vendors’ default settings.^23^ T1-weighted MRI scans were acquired for anatomical reference 1–7 days prior to the PET session at a Philips 3 T Intera system (Philips Healthcare, Best, the Netherlands).^18^

#### [^18^F]florbetapir dataset

Four cognitively normal (CN), Aβ-negative subjects and five AD dementia (Aβ-positive) patients underwent repeated dynamic [^18^F]florbetapir PET scans (average interval: 4.5 ± 3.0 weeks) with arterial blood sampling using a Philips Ingenuity TF PET/CT (Philips Medical Systems, Best, the Netherlands). Prior to the start of a 90 minutes dynamic PET scan, a low-dose CT was acquired. PET images were reconstructed into 22 frames using the BLOB (rotationally symmetric volume elements) ordered-subsets time of flight (BLOB-OS-TF) reconstruction algorithm, with the vendors’ default settings.^24^ T1-weighted MRI scans were acquired on either a Signa HDxt MRI (General Electric, Milwaukee, WI) or an Ingenuity TF PET/MR (Philips Medical Systems, Cleveland, OH) scanner.^19^

#### [^18^F]flortaucipir dataset

A group of 14 participants, consisting of six CN participants (all tau-negative)^25,26^ and eight patients with mild cognitive impairment due to AD or AD dementia (three tau-negative, five tau-positive),^25,26^ underwent repeated dual-time window [^18^F]flortaucipir PET scans (average interval 3.0 ± 1.0 weeks) on a Philips Ingenuity TF PET/CT (Philips Medical Systems, Best, the Netherlands). A low-dose CT was acquired prior to the first phase of the [^18^F]flortaucipir PET scan (0–60 min post injection, p.i.). After a 20-min break, the low-dose CT was repeated and the second phase of the PET scan was acquired (80 to 130 min p.i.). PET images were reconstructed into 29 frames using BLOB-OS-TF reconstruction algorithm. T1-weighted MRI images were acquired <6 months from the PET scan on a 3.0 T Philips Ingenuity Time-of-Flight PET/MR scanner.^25^

#### Arterial sampling procedure

Arterial blood concentrations were measured continuously using an online blood sampler system.^22^ In addition, manual samples were drawn at different times, i.e. at 5.5, 8 and 10 min for [^15^O]H_2_O PET scans and at 5, 10, 20, 40, 60, 75 and 90 min for [^18^F]florbetapir PET scans. These samples were used to calibrate the online sampler curve and, for [^18^F]florbetapir, to estimate plasma-to-whole blood ratios and correct for plasma metabolite fractions. A metabolite corrected plasma input function was obtained after the aforementioned corrections were applied to the continuous online whole blood sampler curve.

### Image processing

During the acquisition of the PET scans, movement was checked regularly using laser beams, and head position was corrected if necessary. A visual quality control was used to assess between-frame motion and scans with severe motion were not included in the present study. Between-frame motion of less than 5 mm was corrected using frame to frame ridged co-registration in VINCI software.^27^ For all scans, T1-weighted MR images were coregistered to their corresponding PET images and the coregistered MR was segmented into grey matter, white matter and cerebrospinal fluid (CSF) using SPM8.^28^ For [^18^F]flortaucipir, the two parts of the PET scan were first combined after coregistering them using VINCI software (Max Plank Institute, Cologne, Germany).^27^ Next, volumes of interest (VOI) were delineated based on the Hammers atlas^29^ as implemented in PVE-lab.^30^ Time-activity curves were obtained for the following grey matter VOIs: medial and lateral anterior temporal lobe, posterior temporal lobe, superior, middle and inferior temporal gyrus, fusiform gyrus, parahippocampal and ambient gyrus, anterior and posterior cingulate gyrus, middle and orbitofrontal gyrus, gyrus rectus, inferior and superior frontal gyrus, pre- and post-central gyrus, superior parietal gyrus and the (infero)lateral remainder of the parietal lobe, and a region consisting of all grey matter voxels of the brain, total grey matter.

### Kinetic modelling

The [^15^O]H_2_O PET dataset had arterial plasma input data available and therefore, the single-tissue compartment model with two rate constants and blood volume fraction parameter was used to estimate *K*_1_, which equals CBF (*F*) as the extraction fraction (*E*) is essentially equal to 100% (*K*_1_ = *E · F*). In addition, to correct for global variations in perfusion, regional *K*_1_ values were normalised by *K*_1_′ (rate of influx of the tracer into the reference tissue) to obtain a measure of relative CBF (*K*_1_/*K*_1_′). For the second dataset consisting of [^18^F]florbetapir PET scans and arterial plasma input data, both the reversible two-tissue compartment model with four rate constants and additional blood volume fraction parameter (2T4k_V_b_) and the simplified reference tissue model (SRTM)^31^ were used to determine *K*_1_, *K*_1_/*K*_1_′ and *R*_1_, respectively. Finally, for [^18^F]flortaucipir PET scans, no plasma input data were available and, therefore, only SRTM was used to derive regional *R*_1_ values.^19^ For all three tracers included in this study, the cerebellar grey matter was used as reference tissue.

### Statistical tests

All statistical analyses were performed in R (version: 4.0.2; R Foundation for Statistical Computing, Vienna, Austria).^32^ Results with *p < *0.05 were considered statistically significant. The rank-biserial correlation *r* for non-parametric tests was used as a measure of effect size because it can be used for between and within study designs.^33–35^ The rank_biserial function from the *effectsizes* package in R was used to compute this measure. Potential between-tracer differences in sex were investigated using a chi-square test, while differences in age were assessed using Kruskal-Wallis^36^ and Mann Whitney U tests.^37^ TrT repeatability was calculated according to equation (1) and, for purposes of visualizing TrT distributions, also non-absolute values were calculated and shown in Violin plots.

(1) $$\mathrm{TrT}\;\mathrm{repeatability}\;\left(\%\right)\;=\;\frac{\left|T-R\right|}{0.5\cdot |T+R|}\cdot 100$$

Next, Bland-Altman analyses^38^ were used to assess whether there was a bias between test and retest measures and to visualize measurement variability. Finally, potential differences in TrT repeatability were assessed for different tracers, metrics, diagnostic groups and groups based on the presence of the target pathology in case of the Aβ and tau tracers. Specifically, Mann-Whitney *U* tests were used for comparing metrics between different tracers and comparisons between diagnostic groups, while Wilcoxon Signed-Rank tests^39^ were used for within tracer comparisons between metrics.

## Results

An overview of the characteristics of the three datasets can be found in Table 1. The only between-tracer difference was in age (χ^2^ = 21.89, *p < *0.001), with the [^15^O]H_2_O dataset consisting of significantly younger participants compared with the other two datasets (both *p < *0.001, *r* = −1.00). Relative CBF values per tracer, diagnostic group and for both test and retest measurements can be found in Supplementary Table 1. Here, it can be observed that for both cognitively normal and cognitively impaired individuals, relative CBF values were comparable between tracers.

**Table 1.** Subject demographics.

	[^15^O]H_2_O	[^18^F]florbetapir		[^18^F]flortaucipir	
	CN (*N *= 11)	CN (*N *= 4)	AD (*N *= 5)	CN (*N *= 6)	MCI/AD (*N *= 8)
Age	22.4 ± 1.7	63.3 ± 2.6	65.0 ± 4.2	64.5 ± 8.8	65.5 ± 9.8
Sex (% female)	45.5%	50%	80%	50%	50%
Injected dose (MBq)					
Test	1100	292 ± 35		237 ± 15	
Retest	1100	312 ± 11		245 ± 18	
Scan interval	21.0 ± 1.0 min	4.5 ± 3.0 weeks		3.0 ± 1.0 weeks	

CN: cognitively normal; AD: Alzheimer’s disease dementia; MCI: mild cognitive impairment. Values are depicted as mean ± SD.

**Figure 1.**
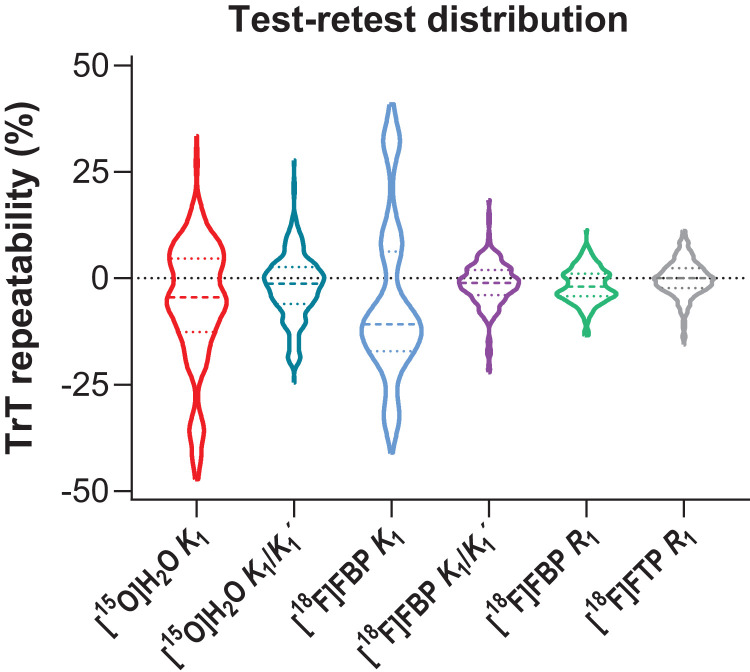
Violin plot showing the distribution of (non-absolute) test-retest values (%) for each tracer and parameter. Dotted coloured lines correspond to quartiles and coloured dashed lines to the median.

**Figure 2.**
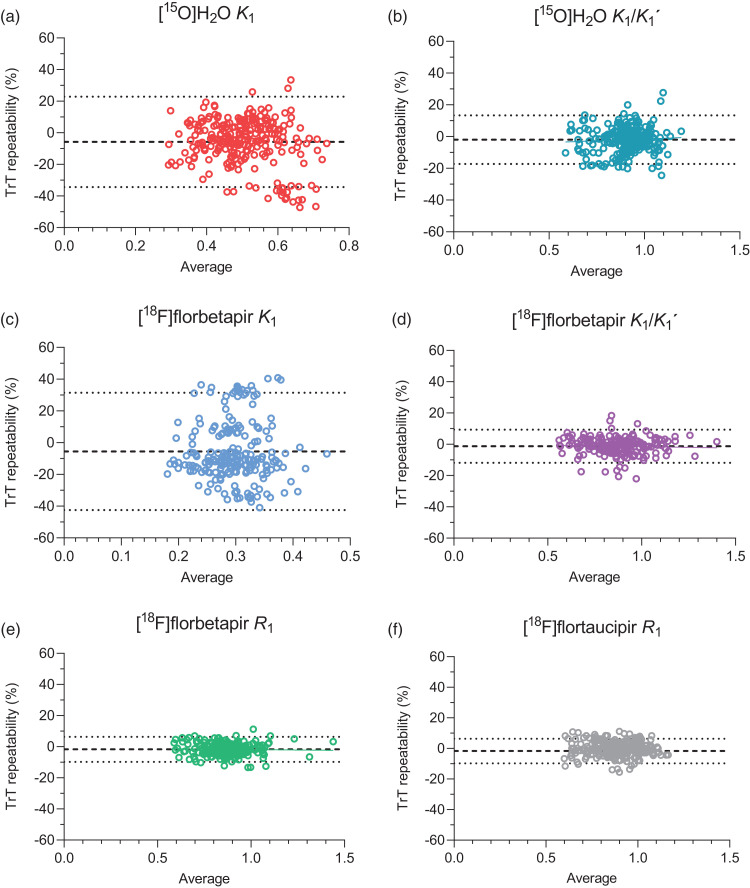
Bland-Altman plots illustrating the test-retest repeatability (%) for (a) [^15^O]H_2_O *K*_1_, (b) [^15^O]H_2_O *K*_1_/*K*_1_′, (c) [^18^F]florbetapir *K*_1_, (d) [^18^F]florbetapir *K*_1_/*K*_1_′, (e) [^18^F]florbetapir *R*_1_ and (f) [^18^F]flortaucipir *R*_1_. Dotted lines correspond to 95% Limits of Agreement and dashed lines to the average % difference.

Figure 1 shows the distribution of (non-absolute) TrT values for each tracer and parameter. Bland-Altman analyses showed that average differences (mean ± SD) between test and retest measures ranged from −0.1 to −2.0% for relative measures: [^15^O]H_2_O *K*_1_/*K*_1_′: −2.0 ± 7.8%, [^18^F]florbetapir *K*_1_/*K*_1_′: −1.3 ± 5.4%, [^18^F]florbetapir *R*_1:_ −1.7 ± 4.1%, [^18^F]flortaucipir *R*_1_: −0.1 ± 4.2,% while for the absolute measure (i.e. [^15^O]H_2_O *K*_1_) it was −5.7 ± 14.6% (Figure 2). The highest variability between test and retest measures was observed for [^15^O]H_2_O *K*_1_. Of note, for [^15^O]H_2_O *K*_1_, a cluster of data points with larger differences between test and retest measures was observed, corresponding to a single subject (Figure 2).

### Test-retest repeatability

#### [^15^O]H_2_O

Across all participants, average TrT repeatability for *K*_1_ was best in the orbitofrontal gyrus 7.8 ± 12.3% and worst in fusiform gyrus 17.7 ± 10.2. For *K*_1_/*K*_1_′, TrT repeatability ranged from 3.1 ± 3.0 in total grey matter to 10.5 ± 6.1 in the fusiform gyrus (Table 2). Overall, TrT repeatability significantly improved for *K*_1_/*K*_1_′ compared with *K*_1_ (*V *= 7510, *p < *0.001, *r* = −0.53).

**Table 2.** Average test-retest repeatability (%) of [^15^O]H_2_O *K*_1_ and *K*_1_/*K*_1_′.

Regions	CBF (*K*_1_)	Relative CBF (*K*_1_/*K*_1_′)
Anterior temporal lobe medial part	14.1 ± 9.5	8.3 ± 5.9
Anterior temporal lobe lateral part	13.7 ± 10.2	6.7 ± 7.0
Parahippocampal and ambient gyri	13.1 ± 8.2	7.0 ± 4.8
Superior temporal gyrus	12.6 ± 14.3	8.0 ± 5.1
Middle and inferior temporal gyri	10.7 ± 10.8	5.2 ± 5.7
Fusiform gyrus	17.7 ± 10.2	10.5 ± 6.1
Insula	11.0 ± 9.7	4.3 ± 4.0
Lateral remainder of occipital lobe	11.9 ± 11.8	8.1 ± 6.3
Gyrus cinguli anterior part	10.5 ± 10.1	5.4 ± 3.3
Gyrus cinguli posterior part	9.6 ± 9.4	4.6 ± 3.8
Middle frontal gyrus	9.4 ± 11.7	5.0 ± 3.1
Posterior temporal lobe	10.9 ± 11.8	4.9 ± 4.3
Inferolateral remainder of parietal lobe	11.2 ± 11.3	3.7 ± 3.4
Precentral gyrus	11.7 ± 8.8	4.9 ± 5.3
Gyrus rectus	13.7 ± 12.5	8.3 ± 5.3
Orbitofrontal gyri	7.8 ± 12.3	4.4 ± 3.6
Inferior frontal gyrus	9.5 ± 13.7	4.0 ± 3.5
Superior frontal gyrus	8.6 ± 10.1	4.5 ± 4.1
Postcentral gyrus	12.0 ± 8.0	5.0 ± 4.0
Superior parietal gyrus	9.9 ± 9.8	4.8 ± 5.1
Lingual gyrus	13.2 ± 11.0	8.7 ± 8.6
Cuneus	13.8 ± 10.7	8.9 ± 8.6
Total grey matter	9.7 ± 10.4	3.1 ± 3.0

All values are depicted as mean ± SD.

#### [^18^F]florbetapir

Across participants, average TrT repeatability *K*_1_ ranged from 14.7 ± 8.0% in the middle and inferior temporal gyri to 19.8 ± 10.0% in the inferolateral remainder of parietal lobe (Supplementary Table 2). TrT repeatability for *K*_1_ was worse in CN (Aβ-negative) subjects compared with AD (Aβ-positive) patients (U = 6419, *p* = 0.008, *r* = 0.21). More specifically, for CN subjects, average TrT repeatability for *K*_1_ ranged from 15.5 ± 4.0% in the middle and inferior temporal gyri to 22.8 ± 12.5% in the posterior part of the gyrus cinguli, while in case of AD patients, TrT repeatability for *K*_1_ ranged from 11.2 ± 9.9% in the parahippocampal and ambient gyri to 20.1 ± 11.9% in the inferolateral remainder of parietal lobe. For *K*_1_/*K*_1_′, average TrT repeatability ranged from 1.4 ± 1.4% in total grey matter to 6.3 ± 6.7% in the orbitofrontal gyrus (Table 3). There was no significant difference in TrT repeatability for *K*_1_/*K*_1_′ between diagnostic groups (*r *= 0.00). For *R*_1_, average TrT repeatability across subjects ranged from 2.1 ± 1.1% in total grey matter to 5.2 ± 2.0% in the fusiform gyrus (Table 4). Overall, TrT repeatability for *R*_1_ was better in CN (Aβ-negative) subjects compared with AD (Aβ-positive) patients (U = 4136, *p* = 0.007, *r* = −0.22). More specifically, for CN subjects, average TrT repeatability for *R*_1_ ranged from 1.0 ± 0.9% in the insula to 6.0 ± 4.4% in the posterior part of the gyrus cinguli, while in case of AD patients, TrT repeatability for *R*_1_ ranged from 1.7 ± 0.9% in the superior temporal gyrus to 6.4 ± 4.0% in the orbitofrontal gyri. There was no significant difference in TrT repeatability between *K*_1_/*K*_1_′ and *R*_1_ (*r *= 0.13), while both measures showed better TrT repeatability than *K*_1_ (U = 20477, *p* < 0.001, *r* = −0.90 and U = 21046, *p* < 0.001, *r* = −0.96, for *K*_1_/*K*_1_′ and *R*_1_, respectively).

**Table 3.** Average test-retest repeatability (%) of [^18^F]florbetapir *K*_1_/*K*_1_′.

Region	All (*N *= 9)	CN (*N *= 4)	AD (*N *= 5)
Anterior temporal lobe medial part	4.2 ± 5.4	3.1 ± 2.6	5.0 ± 7.1
Anterior temporal lobe lateral part	4.7 ± 5.3	3.4 ± 1.9	5.7 ± 7.1
Parahippocampal and ambient gyri	3.5 ± 2.5	1.5 ± 1.4	5.1 ± 1.8
Superior temporal gyrus	3.6 ± 1.5	3.8 ± 2.2	3.4 ± 0.7
Middle and inferior temporal gyri	2.9 ± 2.9	4.6 ± 3.4	1.5 ± 1.6
Fusiform gyrus	3.0 ± 2.6	3.7 ± 2.4	2.5 ± 3.0
Insula	4.4 ± 6.3	2.7 ± 1.0	5.7 ± 8.6
Lateral remainder of occipital lobe	4.2 ± 3.9	2.4 ± 2.4	5.6 ± 4.4
Gyrus cinguli anterior part	5.7 ± 5.0	3.3 ± 1.1	7.6 ± 6.3
Gyrus cinguli posterior part	4.7 ± 2.7	5.1 ± 3.0	4.3 ± 2.7
Middle frontal gyrus	3.7 ± 2.9	3.3 ± 3.7	4.0 ± 2.5
Posterior temporal lobe	4.0 ± 4.7	3.3 ± 2.3	4.6 ± 6.3
Inferolateral remainder of parietal lobe	5.8 ± 5.1	5.1 ± 2.3	6.4 ± 6.9
Precentral gyrus	2.8 ± 1.9	3.2 ± 2.4	2.5 ± 1.6
Gyrus rectus	3.7 ± 4.3	2.5 ± 1.6	4.7 ± 5.6
Orbitofrontal gyri	6.3 ± 6.7	3.5 ± 3.0	8.5 ± 8.3
Inferior frontal gyrus	3.3 ± 3.3	3.3 ± 2.9	3.3 ± 4.0
Superior frontal gyrus	5.4 ± 5.1	3.2 ± 3.9	7.2 ± 5.6
Postcentral gyrus	4.4 ± 2.6	4.3 ± 1.8	4.5 ± 3.3
Superior parietal gyrus	4.0 ± 1.5	5.0 ± 0.5	3.2 ± 1.6
Lingual gyrus	3.4 ± 2.9	4.2 ± 1.4	2.7 ± 3.8
Cuneus	3.0 ± 2.4	2.8 ± 1.7	3.0 ± 3.0
Total grey matter	1.4 ± 1.4	2.1 ± 1.7	0.9 ± 1.0

All values are depicted as mean ± SD; CN: cognitively normal; AD: Alzheimer’s disease dementia.

**Table 4.** Average test-retest repeatability (%) of [^18^F]florbetapir *R*_1._

Region	All (*N *= 9)	CN (*N *= 4)	AD (*N *= 5)
Anterior temporal lobe medial part	3.9 ± 2.6	3.4 ± 0.9	4.4 ± 3.5
Anterior temporal lobe lateral part	4.9 ± 3.2	3.7 ± 1.9	5.9 ± 3.9
Parahippocampal and ambient gyri	3.9 ± 2.6	1.6 ± 0.9	5.8 ± 2.0
Superior temporal gyrus	2.6 ± 2.1	3.8 ± 2.6	1.7 ± 1.0
Middle and inferior temporal gyri	4.5 ± 4.0	5.8 ± 4.9	3.6 ± 3.3
Fusiform gyrus	5.2 ± 2.0	4.1 ± 0.9	6.1 ± 2.3
Insula	2.5 ± 2.5	1.0 ± 0.9	3.6 ± 3.0
Lateral remainder of occipital lobe	2.6 ± 2.2	2.1 ± 1.6	3.1 ± 2.6
Gyrus cinguli anterior part	3.9 ± 2.8	3.4 ± 2.6	4.3 ± 3.2
Gyrus cinguli posterior part	4.5 ± 3.3	6.0 ± 4.4	3.3 ± 1.8
Middle frontal gyrus	4.2 ± 2.0	2.5 ± 1.1	5.6 ± 1.3
Posterior temporal lobe	3.2 ± 2.5	3.5 ± 3.1	3.0 ± 2.3
Inferolateral remainder of parietal lobe	3.8 ± 1.6	3.7 ± 1.3	3.9 ± 1.9
Precentral gyrus	2.5 ± 1.6	1.5 ± 1.2	3.3 ± 1.4
Gyrus rectus	3.7 ± 3.2	3.8 ± 2.4	3.7 ± 4.0
Orbitofrontal gyri	4.7 ± 4.1	2.5 ± 3.5	6.4 ± 4.0
Inferior frontal gyrus	4.0 ± 3.7	2.8 ± 1.6	4.9 ± 4.8
Superior frontal gyrus	4.4 ± 2.4	2.5 ± 1.9	5.8 ± 1.7
Postcentral gyrus	3.4 ± 1.4	3.0 ± 0.8	3.7 ± 1.8
Superior parietal gyrus	3.0 ± 1.9	3.2 ± 1.3	2.8 ± 2.3
Lingual gyrus	2.3 ± 1.7	2.6 ± 2.1	2.0 ± 1.4
Cuneus	2.6 ± 2.1	1.6 ± 1.3	3.4 ± 2.4
Total grey matter	2.1 ± 1.1	1.9 ± 1.0	2.2 ± 1.3

All values are depicted as mean ± SD. CN: cognitively normal; AD: Alzheimer’s disease dementia.

#### [^18^F]flortaucipir

Across participants, average TrT repeatability for *R*_1_ ranged from 1.8 ± 2.0% in total grey matter to 4.3 ± 3.3% in the medial part of the anterior temporal lobe (Table 5). TrT repeatability was better in case of CN participants compared with AD patients (U = 10504, *p* = 0.008, *r* = −0.17). In CN participants, average TrT repeatability for *R*_1_ ranged from 0.6 ± 0.5% in the posterior temporal lobe to 4.8 ± 3.3% in the gyrus rectus. In AD patients, it ranged from 1.4 ± 1.0% in the precentral gyrus to 8.5 ± 8.3% in the orbitofrontal gyri. There were no differences in TrT repeatability between tau-negative and tau-positive groups (*r* = −0.04).

**Table 5.** Average test-retest repeatability (%) of [^18^F]flortaucipir *R*_1_.

Regions	All (*N *= 14)	CN (*N *= 6)	AD (*N *= 8)
Anterior temporal lobe medial part	4.3 ± 3.3	2.9 ± 3.6	5.4 ± 2.6
Anterior temporal lobe lateral part	2.5 ± 2.2	1.7 ± 1.3	3.1 ± 2.6
Parahippocampal and ambient gyri	3.9 ± 3.0	3.0 ± 2.4	4.5 ± 3.2
Superior temporal gyrus	3.3 ± 2.2	2.8 ± 2.6	3.7 ± 1.7
Middle and inferior temporal gyri	2.5 ± 2.2	2.2 ± 1.9	2.7 ± 2.3
Fusiform gyrus	2.5 ± 2.2	1.8 ± 2.0	3.0 ± 2.3
Insula	2.5 ± 2.4	1.0 ± 0.8	3.7 ± 2.5
Lateral remainder of occipital lobe	2.9 ± 2.9	1.2 ± 0.7	4.1 ± 3.3
Gyrus cinguli anterior part	2.8 ± 2.1	2.6 ± 1.3	2.9 ± 2.5
Gyrus cinguli posterior part	2.6 ± 1.8	1.5 ± 1.4	3.5 ± 1.6
Middle frontal gyrus	3.0 ± 1.8	3.5 ± 2.2	2.7 ± 1.2
Posterior temporal lobe	1.8 ± 2.0	0.6 ± 0.5	2.7 ± 2.2
Inferolateral remainder of parietal lobe	3.2 ± 3.3	1.9 ± 1.5	4.1 ± 3.8
Precentral gyrus	1.9 ± 1.1	2.6 ± 0.9	1.4 ± 1.0
Gyrus rectus	4.1 ± 3.0	4.8 ± 3.3	3.5 ± 2.6
Orbitofrontal gyri	3.2 ± 2.6	3.3 ± 2.9	3.2 ± 2.5
Inferior frontal gyrus	2.0 ± 2.0	2.1 ± 2.2	2.0 ± 1.8
Superior frontal gyrus	3.3 ± 2.4	3.7 ± 2.8	3.0 ± 2.0
Postcentral gyrus	2.3 ± 1.3	2.0 ± 1.0	2.6 ± 1.4
Superior parietal gyrus	3.8 ± 3.8	2.1 ± 1.4	5.1 ± 4.4
Lingual gyrus	2.7 ± 2.3	2.7 ± 1.9	2.7 ± 2.6
Cuneus	3.0 ± 2.3	2.1 ± 1.8	3.6 ± 2.5
Total grey matter	1.8 ± 1.3	1.3 ± 1.1	2.2 ± 1.3

All values are depicted as mean ± SD, CN: cognitively normal, AD: Alzheimer’s disease dementia.

#### Tracer comparisons

Between tracer comparisons showed that [^15^O]H_2_O derived *K*_1_ had better TrT repeatability than [^18^F]florbetapir derived *K*_1_ (U = 35851, *p* < 0.001, r = −0.37). Nonetheless, [^18^F]florbetapir derived *K*_1_/*K*_1_′ and *R*_1_ (U = 20323, *p* < 0.001, *r *= −0.22 and U = 20064, *p* < 0.001, *r* = −0.23, respectively), and [^18^F]flortaucipir derived *R*_1_ (U = 27096, p < 0.001, r = −0.33) had better TrT repeatability than [^15^O]H_2_O *K*_1_/*K*_1_′. Furthermore, [^18^F]flortaucipir derived *R*_1_ showed better TrT repeatability than [^18^F]florbetapir derived *R*_1_ (U = 28608, *p* = 0.006, *r* = −0.14).

## Discussion

The present study assessed precision of absolute and relative CBF measures through retrospective analysis of a dynamic [^15^O]H_2_O PET dataset. Furthermore, precision of [^15^O]H_2_O derived absolute/relative CBF was compared with that of (relative) CBF proxies (i.e. *K*_1_, *K*1/*K*_1_′ and *R*_1_) derived from dynamic [^18^F]florbetapir and [^18^F]flortaucipir PET scans.

As expected, for [^15^O]H_2_O PET, TrT repeatability of relative CBF was higher than that of absolute CBF and the same was found for [^18^F]florbetapir. The finding that *K*_1_/*K*_1_′ is less variable than *K*_1_, might be because it contains an intrinsic correction for global fluctuations in CBF,^17^ and/or because any measurement errors in the arterial input function is cancelled out.

Furthermore, better TrT repeatability was observed for [^15^O]H_2_O derived *K*_1_ compared with [^18^F]florbetapir derived *K*_1_. A factor contributing to this finding could be the difference in acquisition time between test and retest [^18^F]florbetapir scans.^40^ For [^18^F]florbetapir and [^18^F]flortaucipir, TrT repeatability of relative CBF (*K*_1_/*K*_1_′ and/or *R*_1_) was significantly better than that of [^15^O]H_2_O PET with a higher effect size for [^18^F]flortaucipir compared with [^18^F]florbetapir. Apart from differences in age and group composition, which could have affected TrT repeatability and will therefore be discussed in more detail later, this finding may be explained by several methodological factors. First, for tracers other than [^15^O]H_2_O, *K*_1_ is not only determined by flow, but also by the extraction fraction (*K*_1_ = *E · F*). Thus, fluctuations in flow might be compensated by changes in extraction fraction. Although higher TrT repeatability (i.e. higher precision) is desirable, it is important to note that this higher repeatability of these relative CBF proxies may, at least in part, be due to dissociation from CBF itself (i.e. by a compensatory change in extraction fraction), which of course is not the case for [^15^O]H_2_O PET. Another plausible explanation for the better TrT repeatability observed with [^18^F]florbetapir and [^18^F]flortaucipir, is the fact that oxygen-15 has a half-life of only 2 min, which means that the total number of counts in the [^15^O]H_2_O scans was much lower than in the [^18^F]florbetapir and [^18^F]flortaucipir scans. This results in higher noise levels in the [^15^O]H_2_O scans, and hence poorer measurement repeatability. The improved TrT repeatability observed for the relative CBF proxies does not necessarily indicate that this measure would be preferred over the gold standard, [^15^O]H_2_O PET. As mentioned above, the relative CBF proxies *R*_1_ or *K*_1_/*K*_1_′ might be biased through changes in extraction fraction, making them less accurate measures than *K*_1_ or *K*_1_/*K*_1_′ derived from [^15^O]H_2_O PET. Furthermore, an important disadvantage of these relative CBF proxies is that, by definition, they can only be used to measure relative changes in flow. Therefore, they cannot be used in research studies that aim to investigate (or are affected by) global changes in CBF. Finally, when designing a research study, it should also be taken into account that *R*_1_ might be less sensitive than the gold standard for measuring changes over time, as reported previously.^11^

In the diagnostic group comparisons, better TrT repeatability was observed for CN participants compared with the cognitively impaired groups for *R*_1_ derived from [^18^F]florbetapir and [^18^F]flortaucipir scans, which might be related to the small datasets used for this comparison and *R*_1_’s relatively small SD. Furthermore, it should be noted that there was a clear difference in age and group composition for the [^15^O]H_2_O PET dataset compared with the [^18^F]florbetapir and [^18^F]flortaucipir datasets, possibly impacting the results. Furthermore, to date, it remains unclear whether TrT repeatability is comparable between young ([^15^O]H_2_O dataset) and elderly ([^18^F]florbetapir and [^18^F]flortaucipir datasets) CN participants, which could also have had an effect on the present results. Evidently, in an ideal scenario, repeatability of all tracers would have been compared within the same participant/patient sample. However, considering ethical regulations regarding radiation exposure of healthy individuals, such a design is not feasible, at least not for the sole reason of comparing *K*_1_ or *R*_1_ repeatability.

Regional differences in TrT repeatability were also observed for all tracers, although no clear pattern could be established. These regional differences may be related to various methodological factors such as size of the region, signal strength, effects of signal spill-in/spill-out from/to adjacent regions, but also biological factors such as atrophy or ageing, considering that these effects are not uniform across the brain.^41,42^ Nonetheless, across tracers, best TrT repeatability of relative CBF was observed for the largest region, which comprised total grey matter. The magnitude of the observed TrT repeatability of *R*_1_ estimated from [^18^F]florbetapir and [^18^F]flortaucipir scans was very comparable to what has previously been reported for [^11^C]PiB, despite the older scanner (Siemens ECAT EXACT HR+) that was used in that study. More specifically, across a nearly identical set of regions and in a group of CN participants, and MCI and AD dementia patients, [^11^C]PiB *R*_1_ TrT repeatability ranged from 1.5 to 5.8%, while in the present study *R*_1_ TrT repeatability was 2.1–5.2% for [^18^F]florbetapir and 1.8–4.3% for [^18^F]flortaucipir. This suggests that despite distinct tracer kinetics and differences in group composition, *R*_1_ remains a stable metric.

Furthermore, it is important to note, that there was a difference in timing between scans in the [^15^O]H_2_O dataset, where all participants received their repeat scan within one day, compared with the [^18^F]florbetapir and [^18^F]flortaucipir datasets, where participants received their repeat scan within a few weeks. Although in a prospective study, timing between repeat scans ideally would have been similar between studies, no changes in CBF are to be expected within a few weeks,^43,44^ thus no considerable effects on the results were expected. Finally, for [^18^F]flortaucipir, no arterial blood data had been collected, which prevented a direct comparison of repeatability of the *K*_1_/*K*_1_′ parameter between all three tracers. However, from a theoretical perspective, no substantial differences were expected in TrT between *K*_1_/*K*_1_′ and *R*_1_, which was also confirmed by comparisons performed using the [^18^F]florbetapir dataset.

## Conclusion

Relative CBF showed higher precision than absolute CBF derived from [^15^O]H_2_O PET scans. Furthermore, relative CBF proxies derived from commonly used amyloid-β and tau tracers appeared to have even higher precision, possibly due to a compensatory effect of extraction fraction. It is important to keep in mind that changes in extraction fraction may bias these proxies, but not the gold standard, [^15^O]H_2_O PET.

## Supplemental Material

sj-pdf-1-jcb-10.1177_0271678X221135270 - Supplemental material for Precision estimates of relative and absolute cerebral blood flow in Alzheimer’s disease and cognitively normal individuals Click here for additional data file. Supplemental material, sj-pdf-1-jcb-10.1177_0271678X221135270 for Precision estimates of relative and absolute cerebral blood flow in Alzheimer’s disease and cognitively normal individuals by Fiona Heeman, Denise Visser, Maqsood Yaqub, Sander Verfaillie, Tessa Timmers, Yolande AL Pijnenburg, Wiesje M van der Flier, Bart NM van Berckel, Ronald Boellaard, Adriaan A Lammertsma, Sandeep SV Golla in Journal of Cerebral Blood Flow & Metabolism
