# Supplementary material for: Precision estimates of relative and absolute cerebral blood flow in Alzheimer’s disease and cognitively normal individuals
Source: J Cereb Blood Flow Metab. 2022 Oct 21;43(3):369–78. doi: 10.1177/0271678X221135270 (PMC9941867; doi:10.1177/0271678X221135270)
Supplement: sj-pdf-1-jcb-10.1177_0271678X221135270 - Supplemental material for Precision estimates of relative and absolute cerebral blood flow in Alzheimer’s disease and cognitively normal individuals [file sj-pdf-1-jcb-10.1177_0271678X221135270.pdf]

Supplementary Table 1. Global cortical relative CBF values per cohort

	$[^{15}O]H_2O$	$[^{18}F]$florbetapir		$[^{18}F]$flortaucipir	
	**CN**	**CN**	**AD**	**CN**	**MCI/AD**
**Test**	0.91 ± 0.04	0.93 ± 0.07	0.86 ± 0.03	0.93 ± 0.04	0.88 ± 0.03
**Retest**	0.92 ± 0.03	0.94 ± 0.07	0.87 ± 0.04	0.92 ± 0.04	0.88 ± 0.04

Relative CBF estimated using K_1/K_1' for H_2O PET and R_1 for $[^{18}F]$florbetapir and $[^{18}F]$flortaucipir PET depicted as mean ± SD. CN: cognitively normal, AD: Alzheimer's disease dementia, MCI: mild cognitive impairment

Supplementary Table 2. Average test-retest repeatability (%) of [^{18}F]florbetapir K_1

**Region**	**All** (*N* = 9)	**CN** (*N* = 4)	**AD** (*N* = 5)
Anterior temporal lobe medial part	15.8 ± 9.6	16.3 ± 10.6	15.5 ± 10.1
Anterior temporal lobe lateral part	17.8 ± 9.1	19.6 ± 8.9	16.3 ± 10.0
Parahippocampal and ambient gyri	15.1 ± 10.8	20.1 ± 11.1	11.2 ± 9.9
Superior temporal gyrus	15.2 ± 9.3	18.0 ± 10.6	13.0 ± 8.8
Middle and inferior temporal gyri	14.7 ± 8.0	15.5 ± 4.0	14.2 ± 10.1
Fusiform gyrus	15.6 ± 10.0	18.7 ± 11.3	13.0 ± 9.2
Insula	15.5 ± 10.8	16.6 ± 11.9	14.6 ± 11.1
Lateral remainder of occipital lobe	15.8 ± 11.7	16.9 ± 13.4	14.8 ± 11.7
Gyrus cinguli anterior part	17.0 ± 11.6	19.7 ± 10.4	14.9 ± 13.3
Gyrus cinguli posterior part	19.2 ± 12.2	22.8 ± 12.5	16.3 ± 12.6
Middle frontal gyrus	18.3 ± 9.1	18.4 ± 8.1	18.2 ± 10.9
Posterior temporal lobe	18.0 ± 9.6	19.2 ± 9.5	17.0 ± 10.8
Inferolateral remainder of parietal lobe	19.8 ± 10.0	19.5 ± 8.6	20.1 ± 11.9
Precentral gyrus	16.2 ± 10.1	16.5 ± 11.0	15.8 ± 10.7
Gyrus rectus	16.0 ± 10.1	19.9 ± 10.7	12.8 ± 9.5
Orbitofrontal gyri	17.7 ± 11.5	15.9 ± 9.8	19.2 ± 13.7
Inferior frontal gyrus	15.7 ± 10.8	17.4 ± 8.8	14.4 ± 13.0
Superior frontal gyrus	17.9 ± 10.3	18.1 ± 8.2	17.8 ± 12.8
Postcentral gyrus	17.8 ± 10.0	16.6 ± 9.7	18.9 ± 11.2
Superior parietal gyrus	17.9 ± 10.3	19.2 ± 10.8	16.9 ± 11.2
Lingual gyrus	15.9 ± 12.7	16.4 ± 16.4	15.4 ± 10.9
Cuneus	14.9 ± 12.8	18.1 ± 15.6	12.3 ± 11.3
Total grey matter	16.9 ± 9.9	18.3 ± 10.5	15.9 ± 10.5

All values are depicted as mean ± SD. CN = Cognitively normal, AD = Alzheimer's disease dementia
